# Coevolution of Age-Structured Tolerance and Virulence

**DOI:** 10.1007/s11538-024-01292-2

**Published:** 2024-04-25

**Authors:** Lydia J. Buckingham, Ben Ashby

**Affiliations:** 1 Department of Mathematical Sciences, University of Bath, Bath, UK; 2 Milner Centre for Evolution, University of Bath, Bath, UK; 3 Department of Mathematics, Simon Fraser University, Burnaby, BC Canada; 4 Pacific Institute on Pathogens, Pandemics and Society, Burnaby, BC Canada

**Keywords:** Host, Pathogen, Parasite, Juvenile, Adult, Age-structure

## Abstract

Hosts can evolve a variety of defences against parasitism, including resistance (which prevents or reduces the spread of infection) and tolerance (which protects against virulence). Some organisms have evolved different levels of tolerance at different life-stages, which is likely to be the result of coevolution with pathogens, and yet it is currently unclear how coevolution drives patterns of age-specific tolerance. Here, we use a model of tolerance-virulence coevolution to investigate how age structure influences coevolutionary dynamics. Specifically, we explore how coevolution unfolds when tolerance and virulence (disease-induced mortality) are age-specific compared to when these traits are uniform across the host lifespan. We find that coevolutionary cycling is relatively common when host tolerance is age-specific, but cycling does not occur when tolerance is the same across all ages. We also find that age-structured tolerance can lead to selection for higher virulence in shorter-lived than in longer-lived hosts, whereas non-age-structured tolerance always leads virulence to increase with host lifespan. Our findings therefore suggest that age structure can have substantial qualitative impacts on host–pathogen coevolution.

**Supplementary Information:**

The online version contains supplementary material available at 10.1007/s11538-024-01292-2.

## Introduction

Pathogens have evolved a wide range of strategies to spread within and transmit between their hosts, often causing significant damage to their hosts in the form of mortality or sterility virulence. Hosts have, in turn, evolved defences against pathogens. Defences may be in the form of resistance, which has an adverse effect on the pathogen (preventing infection and/or subsequent reproduction and transmission of the pathogen) or in the form of tolerance, which does not reduce pathogen fitness (mitigating the negative consequences of pathogen reproduction and transmission for the host). Empirical evidence suggests that tolerance is an important mechanism in protecting a variety of hosts, including plants (Pagan and Garcia-Arenal 2020) and animals (Raberg et al. 2007), from the adverse effects of pathogens. The coevolution of host tolerance and pathogen virulence is therefore likely to have important implications for both epidemiology and evolutionary biology (Little et al. 2010; Seal et al. 2021).

Empirical evidence suggests that host responses to infectious disease vary with host age (Jarosz and Burdon 1990; Sait et al. 1994; Glynn and Moss 2020). However, to measure the level of susceptibility of a host to pathogens, empirical studies often only consider disease-induced mortality or the severity of symptoms of the disease, and so this measure of susceptibility is likely to include components of both resistance and tolerance. However, some empirical work has sought to isolate the effects of resistance and tolerance mechanisms and subsequently looked specifically at age-specific tolerance (Ramsden et al. 2008; Jackson et al. 2014; Regoes et al. 2014; Sorci et al. 2021). Variation in tolerance has been observed between adults and older individuals (Regoes et al. 2014; Sorci et al. 2021) but also between juveniles and adults (Ramsden et al. 2008; Jackson et al. 2014). For example, Sorci et al. (2021) found that mice experienced a significant reduction in tolerance to malaria between the ages of two and twelve months, Regoes et al. (2014) demonstrated that human tolerance to HIV declines with age, and Ramsden et al. (2008) showed that *Drosophila* were more tolerant to bacterial pathogens at three days old than at ten days old.

There are a number of reasons why hosts may experience changes in tolerance as they age. One possible explanation is that the pathogen may inflict different levels of virulence on hosts of different life-stages (see appendix D in Iritani et al. (2019)). This might generate greater selection for tolerance in the life-stage during which the pathogen is more harmful, which could in turn lead to the evolution of age-structured tolerance. Alternatively, hosts may be exposed to more pathogens as juveniles than as adults, or vice versa, perhaps due to differences in social behaviour (Rohani et al. 2010), which may also lead to variation in selection on tolerance at different life-stages. Prior exposure and immune priming may also be factors; older hosts are more likely to have been exposed to the pathogen previously and so may have greater tolerance than younger hosts (Cabrera et al. 2023).

Another possibility is that the host pays different costs for exhibiting tolerance at different life-stages, perhaps due to resource allocation constraints. For example, it may be advantageous to invest in tolerance as a juvenile but not as an adult if that would cause resources to be diverted away from reproduction (Ashby and Bruns 2018; Buckingham et al. 2023). Multiple studies have identified age-specific trade-offs between host defences against disease and reproduction (Chaplin and Mann 1978; Simons 1979; Biere and Antonovics 1996; Tian et al. 2003; Bartlett et al. 2018). For example, Tian et al. (2003) found that a gene which confers protection against a bacterial pathogen in juvenile *Arabidopsis* is also associated with a reduction in seed set (Tian et al. 2003). However, as host defences against the disease were measured by observing symptoms in hosts exposed to the pathogen, this may be indicative of either resistance or tolerance, or a combination of both. Elsewhere, host tolerance specifically has been found to trade off with growth and reproductive traits, with increased tolerance associated with longer pre-reproductive and shorter reproductive periods in plants (Montes et al. 2020). Natural populations also exhibit variation in tolerance (Raberg et al. 2007; Råberg et al. 2009; Henschen et al. 2023), which suggests that it must come at a cost.

There is empirical evidence to suggest that host resistance to pathogens can evolve independently at different life-stages (Bruns et al. 2022), an idea which may also extend to host tolerance (although this has yet to be confirmed empirically). Alternatively, juvenile and adult tolerance traits may be strongly correlated due to shared tolerance mechanisms. Therefore, juvenile and adult tolerance may be independent traits under contrasting selection pressures, or tolerance may be a lifelong trait with no differentiation between the juvenile and adult stages (or juvenile and adult tolerance may be partially correlated). Theoretical models of tolerance evolution generally assume that tolerance is a single, lifelong trait (for example Boots and Bowers 1999; Roy and Kirchner 2000; Restif and Koella 2004; Miller et al. 2005; Best et al. 2014).

In real host–pathogen systems, the pathogen would generally be expected to evolve at least as quickly as the host. It is therefore important to consider coevolution between the host and pathogen, as opposed to evolution only in the host. Coevolution between a pathogen’s intrinsic level of virulence (disease-associated increase in host mortality in the absence of tolerance) and host tolerance (proportional reduction in virulence) has been studied previously in non-age-structured populations (Best et al. 2008, 2010, 2014), including by Best et al. (2014), who found that these models do not typically generate diversity through polymorphism or cycling (Best et al. 2014). The evolution of host resistance in age-structured populations has been theoretically explored (Ashby and Bruns 2018; Buckingham et al. 2023; Buckingham and Ashby 2023), but as far as we are aware, the (co)evolution of age-structured tolerance and virulence has yet to be studied.

In this paper, we theoretically investigate how age-structure affects the coevolution of host tolerance and pathogen mortality virulence. Specifically, we consider the effects of juvenile versus lifelong tolerance on coevolutionary dynamics. Given that tolerance directly offsets virulence, we expect evolution in these traits to be closely linked, with higher virulence typically selecting for higher tolerance, and vice versa. However, it is also possible that high levels of virulence may select against costly tolerance if it is weak or ineffective, or excessive virulence may reduce disease prevalence to the extent that selection for tolerance decreases. We find that which effect dominates depends upon the inclusion or exclusion of age-structure, and that these feedbacks can lead to a variety of coevolutionary outcomes. In particular, we find that coevolutionary cycling only occurs when tolerance is restricted to the juvenile stage and that age-structure can impact upon the qualitative effects of varying lifespan, causing pathogen virulence to be higher in shorter-lived than in longer-lived hosts.

## Methods

### Model Description

We consider a model for the coevolution of host tolerance and pathogen mortality virulence in an asexual, well-mixed host population structured by age into juvenile $\left( J \right)$ and adult $\left( A \right)$ stages. Let $S_i$ and $I_i$ be the densities of susceptible and infected hosts respectively at life-stage $i \in \{ J,A\}$, giving a total host population density of $N = S_J + S_A + I_J + I_A$. Juveniles mature into adults at rate $g > 0$ and adults reproduce at a maximum rate $a > 0$ subject to density-dependent competition given by $q > 0$ (juveniles do not reproduce), giving an overall reproduction rate of $a\left( {1 - qN} \right)$. Juvenile and adult hosts die naturally at rates $b_J$ and $b_A$. Disease transmission is assumed to be density-dependent, with transmission rate $\beta$ and force of infection (rate at which susceptible hosts become infected) given by $\lambda = \beta (I_J + I_A )$. Hosts recover from infection at a constant rate $\gamma$.

Mortality virulence is given by $\alpha \ge 0$ (the disease-associated increase in the host mortality rate in the absence of tolerance) and is reduced due to tolerance by a factor of $\left( {1 - \tau_J } \right)$ and $\left( {1 - \tau_A } \right)$ in juveniles and adults respectively. That is, the overall disease-associated increase in mortality rate at life-stage $i$ is given by $\alpha \left( {1 - \tau_i } \right)$. Note that $0 \le \tau_i \le 1$ and so the host cannot evolve to derive a benefit from its pathogen, nor can it evolve to experience disease-associated mortality greater than the pathogen virulence parameter $\alpha$. As time is measured arbitrarily, rate parameter values can only be interpreted in relation to one another (e.g. comparing $\alpha$ to $b_A$ indicates how many times faster intolerant infected adults die from infection than from other causes).

We seek to determine the effect of age-structured tolerance on the coevolution of tolerance and virulence and so we consider separately the cases where (1) juvenile tolerance evolves with adult tolerance fixed at zero and where (2) juvenile and adult tolerance are equal and evolve as a single trait. This will allow us to consider an age-structured model without the additional complication of three-trait evolution (which would be required if tolerance could evolve independently in juveniles and adults, alongside pathogen evolution).

**Fig. 1:**
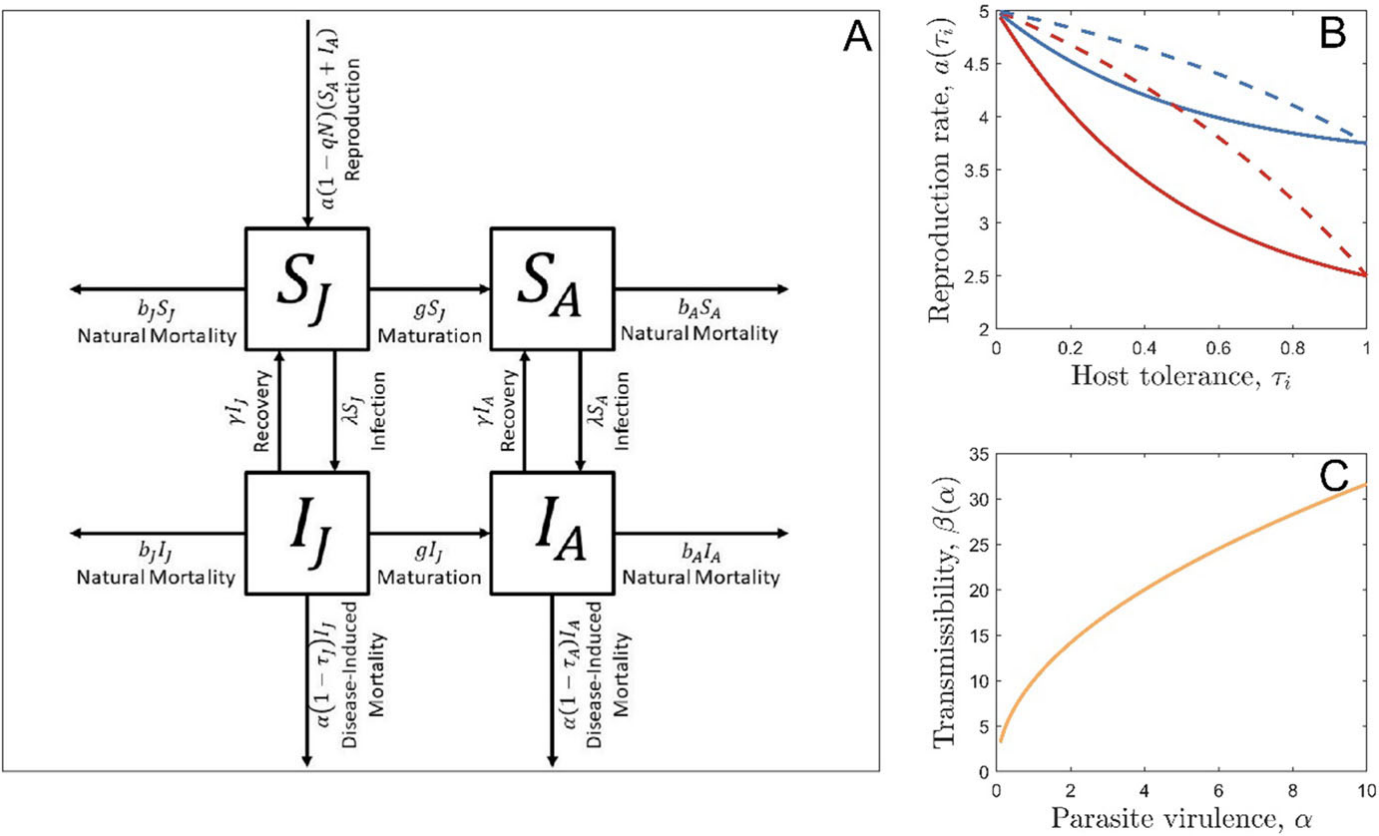
**A** Model schematic for a monomorphic population. **B** Examples of tolerance-reproduction trade-offs (with $a_0 = 5$). Trade-off strength is controlled by the parameter $c_1$; a relatively strong trade-off ($c_1 = 0.5$, red) results in a much larger reduction in the birth rate for a given level of tolerance than a relatively weak trade-off does ($c_1 = 0.25$, blue). Trade-off shape is controlled by the parameter $c_2$; a positive value ($c_2 = 2$, solid) means that the costs decelerate as tolerance increases whereas a negative value ($c_2 = - 1$, dashed) leads to accelerating costs. **C** The virulence-transmission trade-off, with $\beta_0 = 10$ (Color figure online)

In a monomorphic population and in the absence of any costs of tolerance or virulence, the population dynamics are described by the following set of ordinary differential equations (see Fig. 1A for a model schematic and Table 1 for a list of model parameters and variables):

1a $$\frac{dS_J }{{dt}} = a\left( {1 - qN} \right)\left( {S_A + I_A } \right) - \left( {b_J + g + \lambda } \right)S_J + \gamma I_J$$

1b $$\frac{dS_A }{{dt}} = gS_J - \left( {b_A + \lambda } \right)S_A + \gamma I_A$$

1c $$\frac{dI_J }{{dt}} = \lambda S_J - \left( {b_J + g + \alpha \left( {1 - \tau_J } \right) + \gamma } \right)I_J$$

1d $$\frac{dI_A }{{dt}} = gI_J + \lambda S_A - \left( {b_A + \alpha \left( {1 - \tau_A } \right) + \gamma } \right)I_A$$

**Table 1:** Model parameters and variables

Parameter/variable	Description	Default value or range
$a$	Reproduction rate of adult hosts	n/a
$a_0$	Baseline reproduction rate of adult hosts	5
$b_J ,{ }b_A$	Natural mortality rate of juvenile/adult hosts	1/5
$b$	Natural mortality rate of all hosts when $b_J = b_A$	1/5
$c_1$	Strength of tolerance-reproduction trade-off	0.25
$c_2$	Shape of tolerance-reproduction trade-off	3
$g$	Host maturation rate	1
$I_J ,I_A$	Density of infected juveniles/adults	n/a
$N$	Host population density	n/a
$q$	Strength of host density-dependence	1
$S_J ,S_A$	Density of susceptible juveniles/adults	n/a
$t$	Time, measured in arbitrary units	n/a
$\tau_A$	Adult tolerance	0
$\tau_J , \tau_L$	Juvenile/lifelong tolerance	$0 \le \tau_J , \tau_L \le 1$
$\alpha$	Mortality virulence	$0 \le {\upalpha }$
$\beta_0$	Baseline transmission rate	10
$\gamma$	Recovery rate	0 or 1
$\lambda$	Force of infection	n/a
$\phi$	Relative pathogen mutation rate	20

We consider two model versions, one in which tolerance is a lifelong host trait ($\tau_J = \tau_A = \tau_L$) and another in which juvenile tolerance can evolve and adult tolerance is fixed. It is possible to non-dimensionalise this model by rescaling population sizes by $\frac{1}{q}$ and time by $\frac{1}{ \gamma }$; we can therefore fix $q = 1$ and $\gamma = 1$ in Eq. (1) without loss of generality (see *Online Resource*). However, we also consider the case where there is no recovery from infection, in which case $\gamma = 0$. Throughout the paper, we will generally consider the case where juveniles and adults have the same natural mortality rate, $b_J = b_A = b$. When time is rescaled according to the recovery rate, the natural death rate is scaled so that the average host lifespan, $1/b$, is measured in multiples of the average duration of infection.

The host population is viable whenever $ag - b_A \left( {b_J + g} \right) > 0$ and the pathogen is viable whenever the following condition holds (see *Online Resource* for derivation):

2a $$R_0 := \frac{{\beta \hat{S}_J }}{v_J } + \frac{{\beta g\hat{S}_J }}{v_J v_A } + \frac{{\beta \hat{S}_A }}{v_A } > 1$$

where $\hat{S}_J$ and $\hat{S}_A$ represent the disease-free equilibrium of the system and:

2b $$v_J = b_J + g + \alpha \left( {1 - \tau_J } \right) + \gamma$$

2c $$v_A = b_A + \alpha \left( {1 - \tau_A } \right) + \gamma$$

for notational convenience.

In the absence of any costs, the host will always evolve full tolerance and the pathogen will always evolve to zero virulence. We therefore assume that tolerance (juvenile or lifelong) comes at a cost to host reproduction, with the trade-off function given by:

3 $$a\left( {\tau_i } \right) = a_0 \left( {1 - \frac{{c_1 \left( {1 - e^{c_2 \tau_i } } \right)}}{{1 - e^{c_2 } }}} \right)$$

where $i \in \left\{ {J,L} \right\}$ depending on whether the evolving trait is juvenile or lifelong tolerance (trade-off shown in Fig. 1B). We also assume that pathogen virulence is associated with greater transmission, such that:

4 $$\beta \left( {\upalpha } \right) = \beta_0 \sqrt {\alpha }$$

This is a commonly used trade-off function in virulence evolution models (Acevedo et al. 2019) because the pathogen needs to harvest host cells in order to grow within the host and transmit (which causes damage to the host), but this process will likely have diminishing returns for the pathogen (the trade-off is shown in Fig. 1C) (Mackinnon and Read 1999; De Roode et al. 2008).

### Evolutionary Dynamics

The invasion fitness of a rare pathogen mutant can be calculated using the next-generation method (see *Online Resource*) and is given by:

5 $$w_P = \frac{{\beta_0 S_J^* \sqrt {\alpha_m } \left( {b_A + g + \gamma + \alpha_m \left( {1 - \tau_A } \right)} \right) + \beta_0 S_A^* \sqrt {\alpha_m } \left( {b_J + g + \gamma + \alpha_m \left( {1 - \tau_J } \right)} \right)}}{{\left( {b_J + g + \gamma + \alpha_m \left( {1 - \tau_J } \right)} \right)\left( {b_A + \gamma + \alpha_m \left( {1 - \tau_A } \right)} \right)}} - 1$$

where asterisks denote the endemic equilibrium of the system.

The invasion fitness of a rare host mutant is calculated similarly (see *Online Resource*) and is given by:

6a $$w_H = \frac{{a\left( {1 - qN^* } \right)M_1 }}{D} - 1$$

where

6b $$M_1 = gc_J c_A + g\gamma \lambda + g\lambda c_J + g\lambda \left( {b_A + \lambda } \right)$$

6c $$D = \left( {b_J + g + \lambda } \right)\left( {b_A + \lambda } \right)c_J c_A - \gamma \lambda \left( {b_J + g + \lambda } \right)c_J - \gamma \lambda \left( {b_A + \lambda } \right)c_A + \gamma^2 \lambda^2$$

6d $$c_J = b_J + g + \gamma + \alpha \left( {1 - \tau_{J_m } } \right)$$

6e $$c_A = b_A + \gamma + \alpha \left( {1 - \tau_A } \right)$$

for notational convenience.

There is no closed-form analytical expression for the endemic equilibrium of system (1) or for the co-singular strategies. We therefore rely on numerical methods and simulations to determine the evolutionary endpoints of the system. To simulate the coevolution of tolerance and virulence, we first choose resident trait values and an initial population composition, both of which are arbitrary. We solve the ecological dynamics for an arbitrary, fixed time period using an ODE solver and then introduce a new, mutant sub-population of either hosts or pathogens (the individual in the current population which mutates is chosen at random, with pathogens assumed to mutate $\phi$ times faster than hosts, and the mutation is also chosen at random to increase or decrease the current trait value by a small, fixed amount). The ecological dynamics are then solved again for the same fixed time period, after which any sub-population with a sufficiently low density (again, this threshold is arbitrary) is classed as extinct and removed. A new mutant sub-population is then added as before, and these steps are repeated for many evolutionary timesteps until the long-term qualitative behaviour becomes clear.

## Results

We consider the differences between our two model scenarios: lifelong and juvenile-only tolerance. Intuitively, there is a greater benefit to lifelong tolerance than to juvenile-only tolerance (because lifelong tolerance acts for longer) and so, all else being equal, lifelong tolerance will always evolve to be higher than juvenile-only tolerance. For this reason, we focus our attention on qualitative differences between the two scenarios.

We have observed a variety of different coevolutionary outcomes from our model. Tolerance and virulence may both evolve to intermediate levels (at a co-CSS), may rise or fall indefinitely or to their maximum or minimum values, or may cycle indefinitely. Bistability is also possible (for a given set of parameters, we have observed anywhere from zero to three co-singular strategies, including repellers). The stability of these co-singular strategies often depends on the relative mutation rates of the host and pathogen. In particular, convergence stability may be different when the two species have equal mutation rates to when the pathogen mutates sufficiently quickly relative to the host.

### Coevolutionary Equilibria and Runaway Selection

**Fig. 2:**
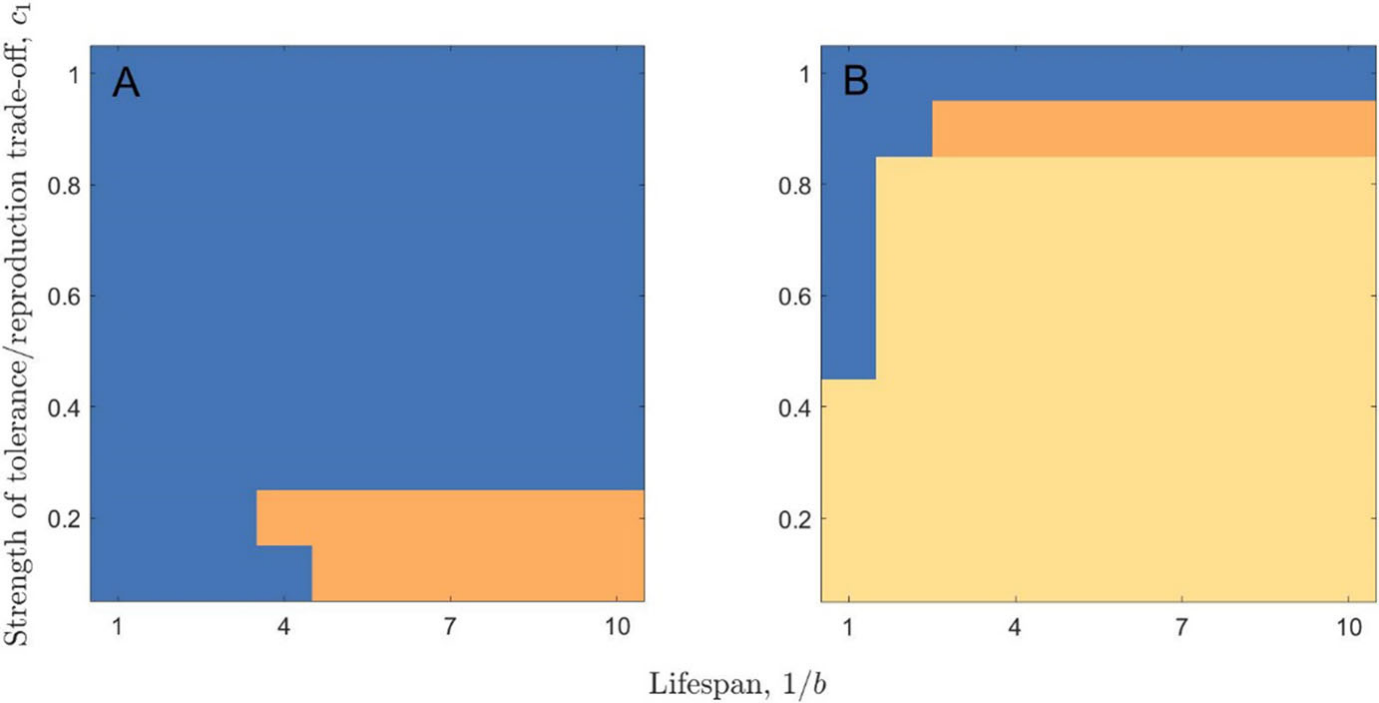
The effect of lifespan and the strength of the tolerance/reproduction trade-off on the incidence of co-CSS’s, bistability and the evolution of full tolerance in the (**A**) juvenile-only and (**B**) lifelong tolerance scenarios. Blue indicates a co-CSS, orange indicates bistability and yellow indicates the evolution of full tolerance and runaway selection for virulence. Parameters used are as in Table 1, except for $\beta_0 = 5$, with $\gamma = 0$ and $\phi = 32$ (Color figure online)

There are a number of similarities between the two model scenarios (when tolerance is lifelong and when tolerance is a juvenile-only trait). In both cases, the host evolves to a stable level of tolerance and the pathogen to a stable level of virulence for a variety of parameter values, but for other parameters bistability (due to the presence of a repeller) can also occur (Figs. 2, S1–S3). However, there is a significant difference between the two model scenarios when the host evolves full tolerance ($\tau_L = 1$). In the lifelong tolerance scenario, this causes the pathogen to experience runaway selection for transmissibility as there is no longer an effective trade-off with virulence (virulence has no negative consequences if the host is fully tolerant throughout its lifetime). If, however, tolerance is a juvenile-only trait (and so adults are never tolerant) or if the costs of tolerance are sufficiently high (and so $\tau_L$ does not evolve to one) then selection for pathogen transmissibility is constrained by the negative effects of virulence. Therefore, runaway selection for increased virulence can only occur in the lifelong tolerance scenario, and only if the cost of high tolerance, $c_1$, is sufficiently low (Figs. 2, S3).

**Fig. 3:**
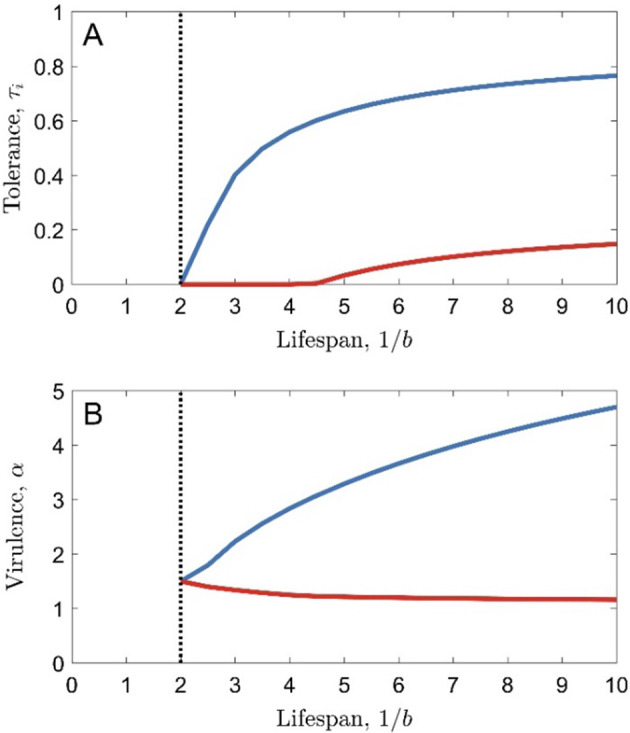
The effect of lifespan on tolerance-virulence coevolution, when tolerance is lifelong (blue curves) or limited to juveniles (red curves). The black, dashed line shows the lifespan below which the pathogen goes extinct. Parameters used are as in Table 1, with $\gamma = 1$ (so lifespan is measured in multiples of the average duration of infection), except for $a_0 = 1$, $c_2 = 4$ and $c_1 = 1$ (full tolerance causes full sterility in the host). Results hold for all host and pathogen mutation rates (all values of $\phi$) (Color figure online)

Tolerance (whether juvenile or lifelong) generally rises with increasing host lifespan (Fig. 3A), all else being equal. This is the result of two factors. Firstly, when hosts have very short lifespans, the infectious period is more constrained and so disease prevalence is low (and hence there is little selection for tolerance); as the host lifespan increases, disease prevalence rises which in turn increases selection for tolerance (Figs. S4, S5A). However, if we vary the baseline pathogen transmissibility, $\beta_0$, alongside lifespan in such a way that the disease prevalence is fixed, then we still see tolerance increase with lifespan, especially in the lifelong tolerance case (Fig. S5B). Therefore, changes in disease prevalence contribute to, but are not solely responsible for, the increase in tolerance with lifespan. Secondly, hosts with longer lifespans have more to gain from surviving an infection because they are less likely to die in any given time period than hosts with shorter lifespans. Hosts with longer lifespans therefore experience stronger selection for tolerance, no matter which model scenario is used.

The effects of lifespan on pathogen virulence, however, are dependent on whether tolerance is lifelong or restricted to juveniles. When tolerance is lifelong, virulence always increases with lifespan, whereas when only juvenile tolerance can evolve, virulence may fall with lifespan (Fig. 3B). This difference arises because of the conflicting effects of lifespan and tolerance on the evolution of virulence. If host tolerance is held evolutionarily static, then pathogen virulence always falls with lifespan (because there is less benefit to the pathogen to reduce its virulence if the host has a shorter lifespan and so is more likely to die soon anyway). However, if host tolerance is under selection, and longer lifespans promote higher tolerance (due to the reasons described above), then higher tolerance should therefore promote the evolution of higher virulence (because tolerance reduces the negative consequences of virulence for the pathogen by preventing the death of the host). Which of these processes dominates will determine whether virulence rises or falls with increasing lifespan. In particular, we would expect that the effect of tolerance on virulence would be stronger when lifelong tolerance evolves than when juvenile tolerance evolves, and so we would expect virulence to rise with increasing lifespan more often in the lifelong tolerance scenario than in the juvenile-only tolerance scenario (e.g. Fig. 3).

### Coevolutionary Cycling

**Fig. 4:**
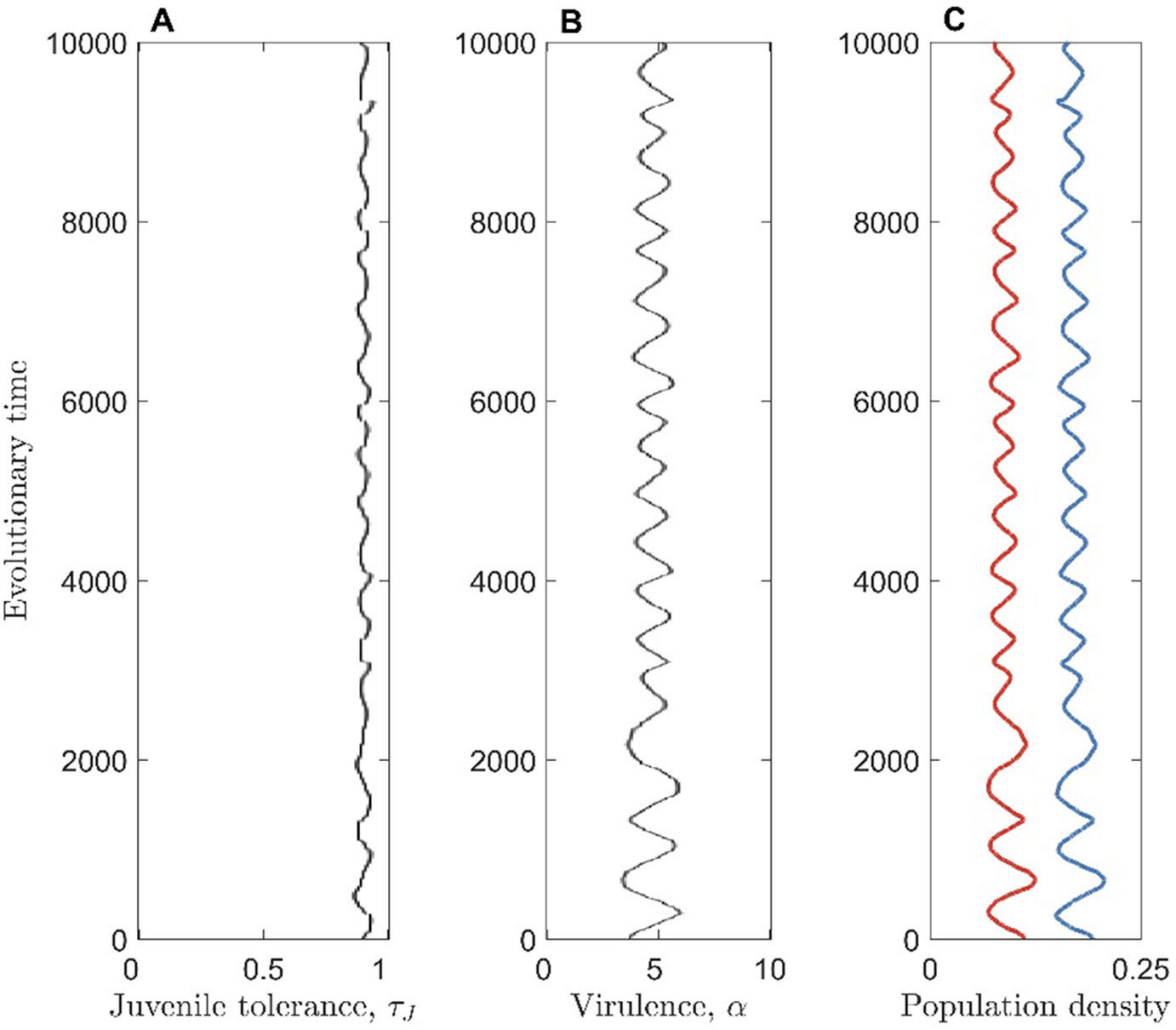
Simulation showing cycling in the host juvenile tolerance (**A**) and pathogen virulence (**B**). The total host (blue) and pathogen (red) population densities are also shown (**C**). Parameters used are as in Table 1, with $\gamma = 0$ (Color figure online)

**Fig. 5:**
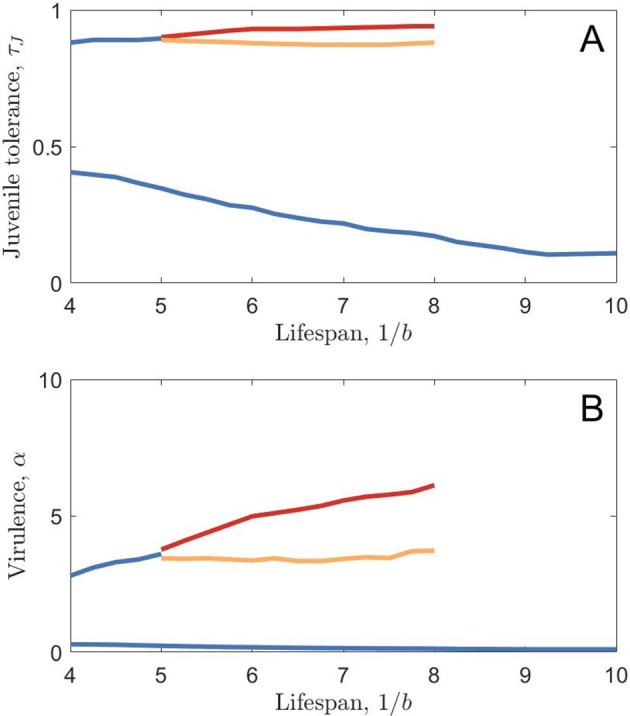
Bifurcation diagram showing the effect of lifespan on cycling. When cycling occurs, red curves show the upper limit of the cycles and orange curves show the lower limit of the cycles. Blue curves show the evolutionary endpoint in the case where no cycling occurs. Parameters used are as in Table 1, with $\gamma = 0$, except for $c_1 = 0.275$ (Color figure online)

As in previous models of tolerance-virulence coevolution (none of which have included age-structure, as far as we are aware), we find that coevolutionary cycling (fluctuating selection dynamics) does not occur when tolerance is a lifelong trait (Fig. S3) (Best et al. 2008, 2010, 2014). However, when tolerance is a juvenile-only trait, coevolutionary cycling is common (Figs. 4, S1–S2), particularly when the host has an intermediate lifespan (Fig. 5) and when the pathogen evolves sufficiently quickly relative to the host (Figs. S1, S6).

Cycling occurs in the juvenile-only tolerance scenario because parasitism initially selects for host tolerance to increase. As tolerance rises, virulence is no longer as detrimental to the pathogen and so selection acts to increase pathogen virulence too. High virulence causes a reduction in disease prevalence, because pathogens are killing their hosts (particularly the adult hosts) more quickly and so have less opportunity to transmit. A reduction in disease prevalence reduces selection for tolerance, which in turn leads to an increase in the cost of virulence for the pathogen. Virulence therefore falls, leading to an increase in disease prevalence, which in turn selects for increased host tolerance. The cycle then begins again. Note that cycling only occurs if higher virulence eventually causes a reduction in disease prevalence. This may seem counterintuitive, as increased virulence is accompanied by increased transmissibility. However, increased virulence also reduces the infectious period and so, unless tolerance keeps pace with virulence, disease prevalence will eventually fall, allowing cycling to occur.

Cycling occurs in the juvenile-only tolerance scenario and not in the lifelong tolerance scenario because increasing pathogen virulence causes a much greater reduction in the density of infected hosts in the juvenile-only tolerance case (because all adult hosts lack tolerance and so are strongly affected by changes in pathogen virulence) (Fig. S7). Although it is possible that cycling may occur when tolerance is a lifelong trait, we have not observed this behaviour despite considering a wide range of parameter sets. This contrasts with the juvenile-only tolerance scenario, where cycling is common. Together with the fact that cycling has not been observed in previous non-age-structured tolerance-virulence coevolution models (Best et al. 2008, 2010, 2014), this suggests that age-structure may be required to generate cycling.

It is worth noting that the cycling we have observed is inherent to this model (not just the effect of stochasticity) and occurs via a Hopf bifurcation as lifespan varies (Figs. S8–S9). Cycling occurs for a wide range of parameter values, but the exact values of lifespan for which it occurs depends heavily on other parameters (Fig. S10). Cycling can occur both when recovery from infection is possible ($\gamma = 1$) and when there is no recovery ($\gamma = 0$), as shown in Figs. S1–S2.

## Discussion

Defences against pathogens and parasites vary with host age and yet many models assume that traits such as tolerance and resistance are consistent throughout the host’s lifetime (Buckingham and Ashby 2022). Here, we examine the effect of lifelong versus age-structured tolerance in a model of tolerance-virulence coevolution. We find that the life stage(s) in which tolerance acts has a significant impact on coevolutionary outcomes. In particular, coevolutionary cycling appears only to occur when tolerance can only evolve in juveniles (rather than as a lifelong trait). Previous models of non-age-structured tolerance-virulence coevolution have never observed such cycling (Best et al. 2008, 2010, 2014), suggesting that age-structure may be essential for generating fluctuating selection dynamics in these models.

The notion that age-structure can induce fluctuating selection is novel, as far as we are aware, and has repercussions for theoretical and empirical work. It suggests, for instance, that theoretical models may be underestimating the prevalence of coevolutionary cycles because of the common assumption that traits are constant throughout the lifespan of the host. This is particularly significant because of the importance of coevolutionary cycling for explaining the evolutionary maintenance of sexual reproduction (Hamilton 1980; Lively 2010; Ashby and Gupta 2014) [but note that coevolutionary cycling is not strictly essential for maintaining sex (Ashby 2020)]. Our findings also suggest that if fluctuating selection is observed empirically, age-related differences in the host may be key drivers of these cycles. This could be tested using time-shift experiments where hosts are only ever infected at a specific life-stage, to see if fluctuating selection occurs.

Age-specific tolerance also qualitatively changes the effects of host lifespan on selection for tolerance and virulence. Under all circumstances, we find that host tolerance rises with increasing lifespan, which concurs with empirical findings (Shukla et al. 2018), as well as with previous theoretical results in non-age-structured populations (Best et al. 2014). However, the effect of lifespan on the evolution of pathogen virulence depends on whether tolerance is age-restricted or lifelong, with virulence generally falling with increasing lifespan when tolerance is limited to juveniles and rising with lifespan when tolerance is lifelong.

Existing theory has generally concluded that virulence should fall with increasing lifespan, as longer lifespans give the pathogen more time to transmit and so lessen selection on high transmission rates (and so on virulence due to the trade-off) (Cressler et al. 2016). However, empirical evidence seeking to test this prediction has yielded mixed results, finding that virulence does indeed fall with increasing lifespan in some systems (Nidelet et al. 2009; Shim and Galvani 2009), but rises with increasing lifespan in others (Ebert and Mangin 1997). This has led to much debate about the factors influencing the effect of lifespan on virulence evolution (Cressler et al. 2016). Our results suggest that two additional factors may be at play. First, coevolution with the host can cause virulence to increase with lifespan (due to an increase in host tolerance with lifespan) and second, age-structure may favour a decrease in virulence with lifespan. These factors are especially important to consider in interventions such as culling, which shortens average host lifespans and may therefore have evolutionary consequences for pathogen virulence (Mennerat et al. 2010).

We focussed our analysis on juvenile versus lifelong tolerance, and did not consider adult-only evolution of tolerance. There is empirical evidence that defences against disease (measured by assessing symptoms of plants exposed to pathogens and therefore reflecting the overall effect of tolerance and resistance strategies) can evolve independently at juvenile and adult stages (Bruns et al. 2022). It is therefore not unreasonable to suppose that juvenile tolerance could evolve without having a significant impact on tolerance at the adult stage. However, it may be more realistic to allow adult tolerance to evolve as well. Our model could be extended in this way to consider the coevolution of three traits: juvenile and adult tolerance and pathogen virulence. This would complicate the analysis considerably but would provide a more general picture of the effect of age-structured tolerance.

Our model has assumed a universal system of infection genetics: all pathogens can kill all hosts; pathogens with a high value of $\alpha$ are universally more virulent than those with a low value of $\alpha$, no matter what host they are infecting; and hosts with a high value of $\tau_i$ are universally more tolerant than those with a low value of $\tau_i$, no matter what pathogen they are infected by. However, many host–pathogen systems exhibit genetic specificity, where some pathogens are better at infecting some hosts than others (Antonovics et al. 2002; Dybdahl et al. 2014). Our model could readily be extended to consider how specificity mediates selection for tolerance and virulence.

In this paper, we have considered only tolerance as a mechanism of host defence against pathogens. However, both tolerance and resistance play important roles in shaping host–pathogen interactions (Schneider and Ayres 2008). Non-age-structured, three-trait coevolution of pathogen virulence, host tolerance and host resistance has been modelled previously (Carval and Ferriere 2010), but this has never been approached in an age-structured context. Carval and Ferriere (2010) found that three-trait coevolution led to higher virulence and lower tolerance than when only one of resistance or tolerance could evolve in the host. Adding an evolvable host resistance trait to our model may have a similar quantitative effect, but the implications for our qualitative results are unclear. We have also only considered the case of mortality virulence and mortality tolerance. However, many pathogens reduce the fecundity of their hosts, either instead of or in addition to increasing mortality. Our model could be adapted to consider the coevolution of sterility virulence and sterility tolerance instead of, or in addition to, mortality virulence and mortality tolerance.

Our model incorporated two trade-offs: one between pathogen virulence and transmissibility and another between host tolerance and reproduction. Several empirical studies have found direct or indirect (via pathogen load) relationships between pathogen virulence and transmission (De Roode et al. 2008; Blanquart et al. 2016; Hawley et al. 2023), and these have inspired the virulence-transmission trade-offs used in many models of pathogen evolution, including our model. The trade-off between host tolerance and reproduction was chosen based on empirical evidence for the existence of trade-offs between disease resistance and reproduction in plants (Chaplin and Mann 1978; Simons 1979; Tian et al. 2003). However, it is possible that tolerance may come at a cost to other host life-history traits, such us maturation ($g$) or mortality ($b$). We have previously shown that the specific traits involved in trade-offs with resistance can have significant impacts on evolutionary outcomes (Buckingham et al. 2023) but whether this holds for tolerance remains to be seen.

Overall, we have shown that the life-stage(s) at which tolerance acts can have a significant impact on host–pathogen coevolutionary dynamics, in particular leading to coevolutionary cycling in tolerance and virulence when tolerance only occurs at the juvenile stage. Our findings further highlight the importance of age-structure in mediating host and pathogen evolutionary outcomes.

### Supplementary Information

Below is the link to the electronic supplementary material.

Supplementary file1 (PDF 966 kb)

## Data Availability

Source code is available in the GitHub repository at: https://github.com/ecoevotheory/Buckingham_and_Ashby_2024
